# Probing the Hydrophobic Region of a Lipid Bilayer at Specific Depths Using Vibrational Spectroscopy

**DOI:** 10.1021/jacs.3c10178

**Published:** 2023-11-20

**Authors:** Md Muhaiminul Islam, Sithara U. Nawagamuwage, Igor V. Parshin, Margaret C. Richard, Alexander L. Burin, Igor V. Rubtsov

**Affiliations:** Department of Chemistry, Tulane University, New Orleans, Louisiana 70118, United States

## Abstract

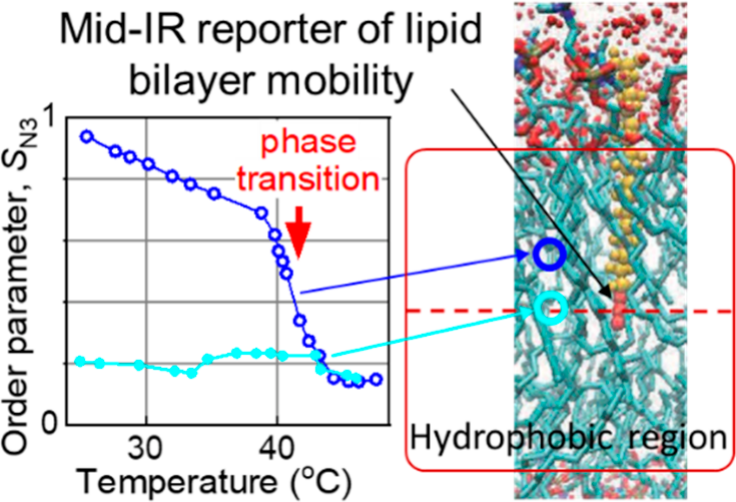

A novel spectroscopic approach for studying the flexibility and mobility in the hydrophobic interior of lipid bilayers at specific depths is proposed. A set of test compounds featuring an azido moiety and a cyano or carboxylic acid moiety, connected by an alkyl chain of different lengths, was synthesized. FTIR data and molecular dynamics calculations indicated that the test compounds in a bilayer are oriented so that the cyano or carboxylic acid moiety is located in the lipid head-group region, while the azido group stays inside the bilayer at the depth determined by its alkyl chain length. We found that the asymmetric stretching mode of the azido group (ν_N3_) can serve as a reporter of the membrane interior dynamics. FTIR and two-dimensional infrared (2DIR) studies were performed at different temperatures, ranging from 22 to 45 °C, covering the Lβ–Lα phase transition temperature of dipalmitoylphosphatidylcholine (∼41 °C). The width of the ν_N3_ peak was found to be very sensitive to the phase transition and to the temperature in general. We introduced an order parameter, *S*_N3_, which characterizes restrictions to motion inside the bilayer. 2DIR spectra of ν_N3_ showed different extents of inhomogeneity at different depths in the bilayer, with the smallest inhomogeneity in the middle of the leaflet. The spectral diffusion dynamics of the N_3_ peak was found to be dependent on the depth of the N_3_ group location in the bilayer. The obtained results enhance our understanding of the bilayer dynamics and can be extended to investigate membranes with more complex compositions.

## 1 Introduction

A cell membrane is one of the major components of a cell and is crucial for living organisms. It provides a functional barrier between the cell and the external environment. But its role is much more complex than a physical barrier and involves several other major functions such as providing a fixed environment for the cell, transporting external substances, interacting with the other cells via proteins, and others.^1,2^ The environment inside the lipid bilayer directly influences the functions of the membrane proteins^3−8^ and hence impacts cell signaling, defense mechanisms, and other biological functions.^9^ Therefore, studies of membrane structure and dynamics are fundamentally important for understanding biological systems. There are hundreds of types of membrane lipids that form a common bilayer structure, which confers specific properties on membranes.^10,11^ The variety in lipid composition induces specific lipid–lipid and lipid–protein interactions.^12,13^ There have been significant efforts to understand how the structural and chemical diversity of lipids affect membrane properties.^14−20^ However, elucidating the dynamic landscape of a lipid bilayer with both high spatial and temporal resolutions still poses significant challenges.

Various techniques have been used to study the complex environment of the lipid bilayer interior, including fluorescence microscopy, NMR spectroscopy, atomic force microscopy, ESR spectroscopy, vibrational spectroscopy, and X-ray and neutron scattering.^10,11,19,21−26^ A large body of data were obtained using NMR spectroscopy, focusing on structural features of the fatty-acid chains of the lipids (CH bond angle distribution) and dynamics of the interior slower than 100 ps.^24,27,28^ The CH bond angular distribution with respect to the normal of the bilayer was expressed in the form of an order parameter, which was found to be depth dependent inside the bilayer. High chain order was found in the middle of a single leaflet; the order parameter decreases slightly toward the carbonyl groups of the lipid but decreases sharply toward the middle of the bilayer.^29,30^ Molecular dynamics (MD) simulation was used actively to understand the properties of the bilayers, supporting NMR studies.^24,27,31−33^ Other experimental methods, such as ESR and fluorescence, also observed depth-dependent changes of the order inside the bilayer, although the methods used bulky probes, which can perturb the bilayer significantly.^34,35^ Nevertheless, to date, direct experimental approaches capable of probing the dynamics of the cell membrane interior with high site selectivity and high temporal resolution are lacking.

Vibrational spectroscopy can offer small probes and high temporal resolution. C–H stretching modes of methylene groups were found to be useful to probe lipid conformations in lipids, allowing to discriminate between gauche- and anti-conformations.^36−39^ However, atomic assignment using CH stretching modes is difficult due to vibrational coupling of different methylene groups along the chain. Two-dimensional infrared (2DIR) spectroscopy has emerged as a tool for elucidating molecular interactions and structural dynamics, including those of the lipid bilayers.^40−45^ Molecular motions of a cell membrane, including the motions on an ultrafast scale, are essential for the cell to conduct its biological function. Vibrational modes are highly sensitive to the local environment, and fluctuations in the environment can be detected by using a 2DIR spectral diffusion method, which can use either natural vibrational modes or external IR probes. While most ultrafast studies on lipid bilayers have focused on the head-group region due to the availability of strong and localized vibrational modes, these studies have provided significant insights into the lipid membrane dynamics.^45−55^ Fayer and co-workers identified the existence of two major types of water molecules in this region,^51^ while Tokmakoff and co-workers characterized the electric field fluctuations at the interfacial region of the lipid bilayer.^53^ Temperature jump spectroscopy was also used to extend the experimentally observable time beyond the vibrational lifetime and investigate the response of membrane proteins to structural changes during lipid phase transitions.^52,54^ Additionally, Righini and co-workers used chain-specific isotopic labeling of the ester carbon to show that the inhomogeneous character of the optical response is determined by inter- and intramolecular electrostatic interactions.^56,57^

Lipid bilayers bahave as a dynamic solvent bath for chemical processes of proteins and other membrane biomolecules. Local protein crowding also affects localized diffusion in lipid bilayers.^19^ However, a clear picture of localized dynamics in the hydrophobic region is still lacking due to the difficulty of introducing small-sized structural reporters at specific depth in a bilayer. Nonetheless, some studies have shed light on the hydrophobic region. Fayer and co-workers used a W(CO)_6_ test compound inside the bilayer and found that the structural dynamics of curved vesicle bilayers are faster compared to planar bilayers, suggesting that the geometry of the membrane plays a significant role in the bilayer dynamics.^43^ Moreover, their findings indicate that the dynamics in the hydrophobic region of the bilayer are largely independent of the hydration level and cholesterol concentration, except for a sudden change occurring between 25% and 35% cholesterol concentration, which suggests a sudden structural transition with changes in cholesterol content.^42,44^

The Rubtsov group recently developed a test compound featuring a small-sized azido group to probe dynamics inside the hydrophobic core at specific depths.^58^ It was shown that the test compound containing azido and cyano end groups linked by an 11-carbon alkyl spacer N_3_(CH_2_)_11_CN (denoted as az11CN) is capable of probing the mobility of the lipid membrane interior.^58^ It was shown that the compound was oriented in such a way that the azido group was in the nonpolar tail region while the cyano group was close to the polar head-group region. Note that the azido group has a strong absorption peak at ca. 2100 cm^–1^ located in a convenient spectral region free from the strong absorption of water and most other compounds. In addition to having a high extinction coefficient and transition dipole, the azido group features high sensitivity to the solvent environment, making it a powerful probe for biomolecular dynamics.^58−66^

**Figure 1:**
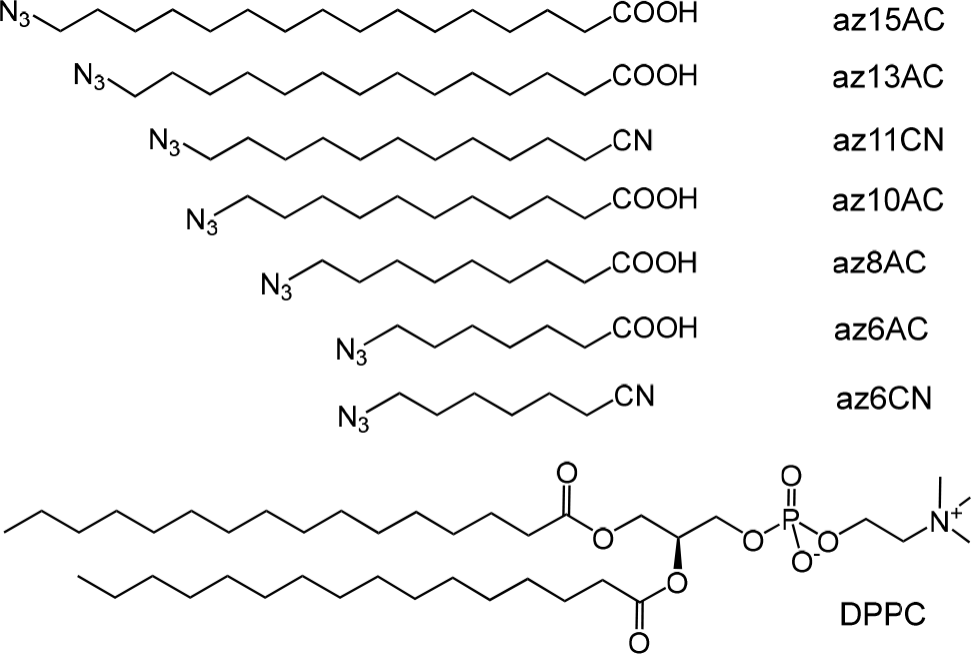
Structures of the test compounds and the DPPC lipid.

In this study, we investigate how sensitive the test compounds of the N_3_-(CH_2_)_*n*_CN and N_3_-(CH_2_)_*n*_-COOH types are to changes in the environment inside the membrane. To investigate the depth-dependent properties of a dipalmitoylphosphatidylcholine (DPPC) membrane, we prepared a set of compounds with different alkyl chain lengths (*n* = 6, 8, 10, 11, 13, 15), while either the cyano or carboxylic acid moiety served as a polar group (Figure 1). We performed FTIR and 2DIR measurements observing the asymmetric stretching mode of the azido moiety, ν_N3_, in compounds with different *n* and at temperatures below and above the lipid phase transition. Phase transitions in lipid bilayers are critical phenomena that heavily influence the behavior and properties of biological membranes. The temperature at which these transitions occur depends on the lipid chain length and the number of double bonds within the lipid molecules. In cellular membranes, both ordered and disordered lipid phases coexist, providing a dynamic solvent environment for proteins.^18^ Understanding the changes associated with the phase transitions has been a major focus in the study of biological membranes, as they have a profound impact on membrane dynamics and protein functionality. In addition, the transition from an ordered gel phase to a disordered fluid (liquid crystalline) phase results in a decrease in membrane thickness, which can subsequently modulate protein functions.^67,68^ The following questions are targeted in this study: Is the azido group of test compounds featuring different lengths of the alkyl linker actually located at different depths in the bilayer? If so, can the azido-group peak, ν_N3_, report on the bilayer mobility at different depths? To answer these questions, we performed spectroscopic measurements at different temperatures to attempt to monitor the phase transition of the bilayer at different depths. MD computations were performed to better understand the locations of the test compounds in the bilayer.

Note that the targeted goals are not achievable if the N_3_ group would behave as a typical IR label showing sensitivity of its central frequency to the local polarity of the environment, as the polarity of the lipid bilayer interior is nonpolar and changes little with the depth in the bilayer. Unique properties of the N_3_ label allowed us to address the above questions.

## 2 Experimental Details

### Sample Preparation and Spectroscopic Measurements

A set of test compounds (Figure 1) was synthesized according to the reported procedure (see the Supporting Information (SI)).^58^ The experiments were performed with planar multilamellar bilayer (MLBL) samples of DPPC (Avanti Polar Lipids) with an incorporated guest compound at ca. 1:10 guest-to-lipid molar ratio, prepared by the isopotential spin-dry ultracentrifugation method.^34^ The sample thickness was about 50 μm. The water content was maintained at a constant level of 13–15 water molecules per lipid; the same results were found with the water content exceeding 10 water molecules per lipid. The experiments were also conducted with a 1:20 guest/lipid molar ratio, and no differences were found. Therefore, the aggregation of the guest molecules in the bilayer can be neglected.

For spectroscopic measurements, the MLBL sample was placed into a sealed cell with 1-mm-thick CaF_2_ windows. To ensure precise temperature control during spectroscopic measurements, an insulating jacket was used to enclose the sample cell, which was equipped with a temperature controller. The temperature was kept constant (±0.3 °C) throughout the measurements and monitored by using a thermocouple.

2DIR spectra were measured using a fully automated dual-frequency three-pulse photon echo spectrometer described elsewhere.^69,70^ Absorptive 2DIR spectra were recorded at different waiting times, *T*_w_, the time between the mode excitation and detection (see the SI for details).

## 3 Results and Discussion

### 3.1 Linear Absorption Spectra of the Test Compounds in a Bilayer

**Figure 2:**
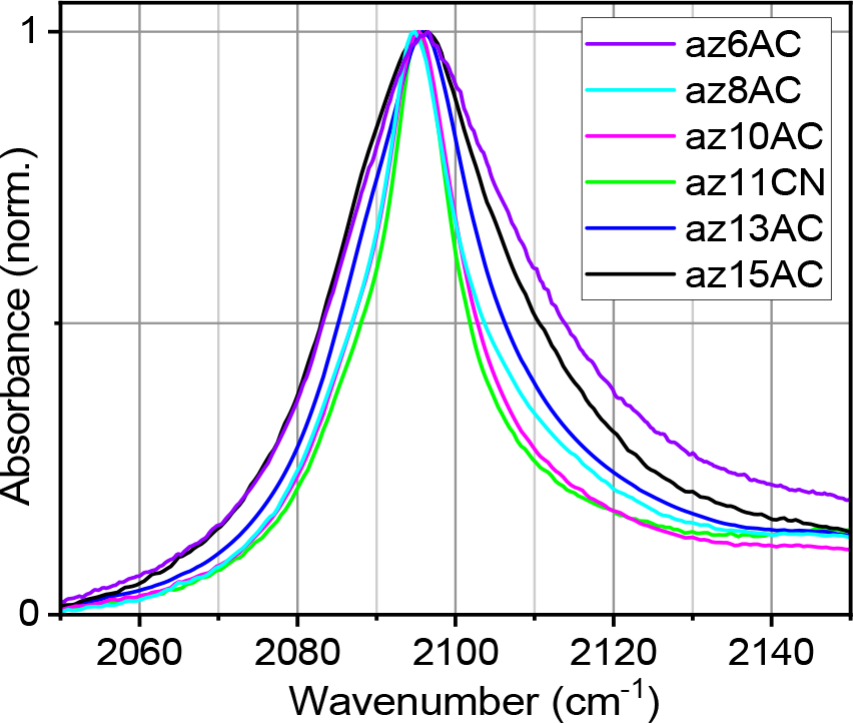
Normalized, background-subtracted FTIR absorption spectra of the N_3_ asymmetric stretching mode of indicated test compounds in the DPPC bilayer.

The absorption spectra of the N_3_ asymmetric stretching mode (ν_N3_) of different test compounds in a DPPC bilayer differ greatly in the peak width, while the central frequencies are similar at ca. 2096 cm^–1^ (Figure 2). The central frequency is consistent with the central frequency of the labels in nonpolar solvents, found at ca. 2096.2 cm^–1^ in hexadecane,^58,65^ indicating that the N_3_ moiety for all the test compounds is located in the hydrophobic region of the bilayer. The full width at half-maximum (Δν_fwhm_) values of the ν_N3_ peak of different test compounds in MLBL span from 15 to 28 cm^–1^ at room temperature (Figure 2). However, in nonpolar solvents, such as hexadecane, all of the test compounds show a similar ν_N3_ spectral width of ca. 26.7 cm^–1^.

**Scheme 1:**
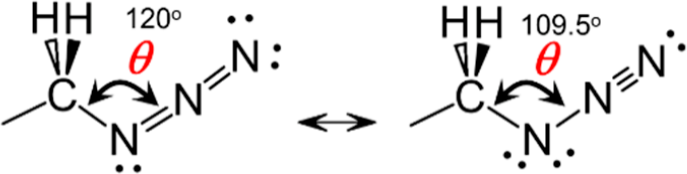
Resonant Lewis Structures of the −CH_2_–N_3_ Moiety

The difference in Δν_fwhm_ values for the test compounds of different chain lengths suggests that their N_3_ labels are indeed located at different depths in the hydrophobic region of the bilayer. Note that, while the azido group peak width does increase with the solvent polarity, reaching 35.5 cm^–1^ in methanol,^58,65^ the width is already very large in nonpolar solvents. It has been shown that the large width of ν_N3_ in azido alkyls is caused by several effects that are unique for the N_3_ group. First, there is a strong Fermi resonance for the ν_N3_ mode, which is coupled to the combination band of the N_3_ symmetric stretching and C–N stretching modes.^58,71,72^ The coupling is strong, resulting in the intensity borrowing from the ν_N3_ fundamental state to the combination band. Second, the potential energy surface along the CNN angle, θ, is very soft, explained by the presence of two resonance structures (Scheme 1), featuring different θ angles. The computed range of θ angles for which the energy of the compound stays within *k*_B_*T* is ca. 11 degrees;^58^ here *k*_B_ is the Boltzmann constant, and *T* is the temperature. Compounds with different θ angles feature different frequencies of the Fermi resonance peaks, resulting in a severely broadened ν_N3_ absorption peak.^58^ Moreover, when the azido group is attached to an alkyl group, the number of Fermi resonances grows significantly as the local N_3_ symmetric stretching mode is harmonically mixed with the CH_2_ wagging modes of the delocalized alkyl chain wagging band and the CN stretching mode is mixed with the modes of the CH_2_ rocking band.^72−74^ The resulting spectrum is not Gaussian in shape but has a shoulder at the high-frequency side (Figure 2). Note that the whole spectrum is greatly affected by numerous Fermi resonances, resulting in exceptional broadening due to the softness of the θ angle. The computations suggest that, if the allowed θ angles are restricted by the environment, the ν_N3_ peak should become narrower.^58^

The strikingly large range of peak widths of ν_N3_ in test compounds featuring different alkyl chain lengths suggests that the bilayer microenvironment of the N_3_ moiety differs greatly at different depths. The data shown in Figure 2 indicate that when the N_3_ group is located near the middle of a single leaflet (C8–C11 chain length), it is experiencing the strongest structural restrictions for its motion, resulting in the narrowest ν_N3_ peak (Figure 2). Such a narrow width indicates that the environment is highly ordered and tightly packed near the azido group of the test compounds with C8–C11 chain lengths, which restricts the θ angle distribution, reducing the peak width. Interestingly, the peak width increases monotonically when the N_3_ label is placed deeper into the bilayer, reaching 28 cm^–1^ for az15AC, for which the N_3_ label is close to the center of the bilayer, the region where two leaflets meet.

The N_3_ peak width is large for the az6AC compound, where the N_3_ label is close to the carbonyl region of the lipids. It is expected that such a short alkyl chain provides a smaller stabilization energy for the alignment of the test compound parallel to the lipid alkyl chains, which may result in a wider depth distribution of the N_3_-group locations, *vide infra*.

### 3.2 Temperature Dependence of the N_3_ Label Line Width

Note that at room temperature the DPPC bilayer exists in the gel (microcrystalline) state, where the long alkyl chains of the lipid feature predominantly all-anti-conformations. Such an ordered state can be detected by observing a characteristic peak of the CH_2_ symmetric and asymmetric stretching modes at ca. 2920 and 2850 cm^–1^.^36,75^ The Lβ–Lα phase transition from the ordered gel state to the more disordered liquid crystalline state occurs at ca. 41 °C for DPPC. In the disordered state at elevated temperatures, the alkyl chains of the lipid feature a much larger number of gauche kinks. We observed the phase transition in our multilamellar bilayer samples with embedded guest molecules using the peak at 2920 and 2850 cm^–1^ and confirmed that it occurs at *T*_ph_ ∼ 41 °C, as expected (Figure S1). Note that the CH_2_ stretching modes of the lipids report on the depth-averaged not depth-specific ordering of the lipid bilayer.

**Figure 3:**
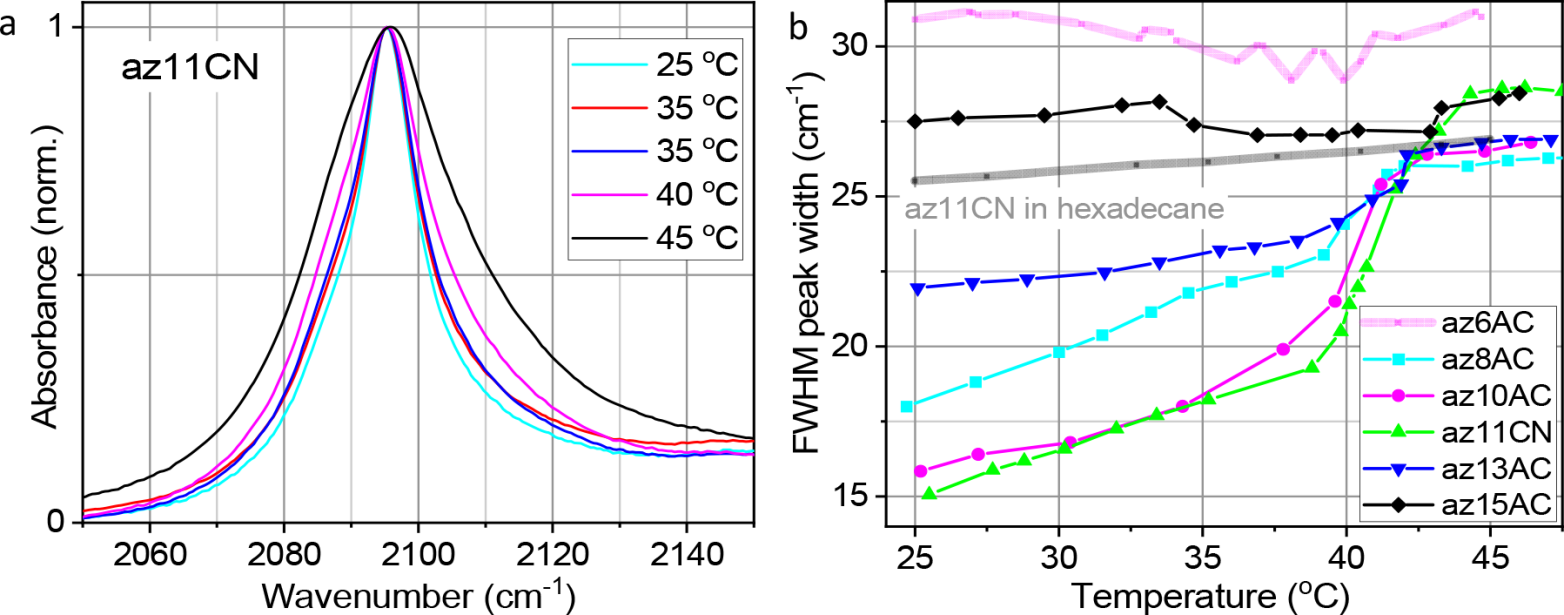
(a) Normalized, background-subtracted FTIR absorption spectra of ν_N3_ of az11CN in the DPPC bilayer at indicated temperatures. (b) ν_N3_ peak width as a function of temperature for indicated compounds in the DPPC bilayer. Gray line shows the width of ν_N3_ for az11CN in a hexadecane solution.

To test if the azido label of the test compounds is capable of reporting on depth-specific microenvironment in the lipid bilayer, we measured temperature dependences of the N_3_ peak width for the test compounds of different alkyl chain length, *n*, varying the temperature across the phase transition. We hypothesize that the chain length determines the depth of the N_3_ label in the bilayer in a monotonic fashion, which will be tested by using the experimental data and MD simulations (*vide infra*). Figure 3A shows the normalized FTIR absorption spectra of ν_N3_ for az11CN in DPPC at different temperatures. With the temperature increase, the central frequency of the peak does not change much, while the peak width changes drastically. The temperature dependence of the N_3_ peak width (Δν_fwhm_) is shown in Figure 3B for the compounds indicated in the inset. For az11CN (green triangles), the peak width grows slowly from 25 to 39 °C and then grows sharply at the temperature of the phase transition (∼41 °C). The width changes are small after the phase transition (>44 °C).

The temperature dependences for all test compounds, except for az15AC and az6AC, are similar in shape. They feature similar peak widths at the temperatures above the phase transition (27–28 cm^–1^), step-like width changes at the phase transition temperature, although the step sizes are different, and monotonic width increases when the temperature is raised from room temperature to 39 °C. The width of ν_N3_ for az11CN in hexadecane solution increases little with temperature over the studied temperature range (Figure 3b, gray line), likely affected by an increased homogeneous width and conformational distribution with temperature. Similar behavior is found for other test compounds and for other solvents (Figure S2). Importantly, the test compounds with *n* = 8–13 are capable of tracking the phase transition inside the lipid membrane. The difference in the temperature dependences for compounds with *n* = 8–13 indicates that the azido labels in different test compounds are indeed located at different depths in the bilayer and that the packing order varies with the depth in the bilayer. The temperature traces indicate that the highest packing order in the bilayer is found at the depth probed by test compounds with *n* = 10 and 11, as these test compounds show the narrowest room-temperature peak width and the largest width increase at the phase transition temperature.

The room-temperature width for az15AC in the bilayer is large, and its temperature dependence shows only small changes, suggesting high disorder near the middle of the bilayer already at room temperature. The shortest test compound, az6AC, features a ν_N3_ width at room temperature that is larger than that in the hexadecane solvent by ca. 5 cm^–1^, suggesting that the azido group of az6AC is exposed to the environment with higher polarity and is located close to the carbonyl groups of the lipids. The width of az6AC decreases with temperature by ca. 2 cm^–1^ and then grows again, showing a resemblance of the phase transition at 41 °C. The data suggest that the N_3_ labels of different test compounds are indeed located at different depths, *z*(*n*), and that the depth increases monotonically with the alkyl chain length, *n*.

Note that the depth of the N_3_ label is determined by the competition of several key interactions. The polar COOH or CN group prefers the polar environment of the lipid head groups, while the nonpolar alkyl chain has minimal energy when embedded into the hydrophobic portion of the bilayer. The azido group prefers the region of medium polarity associated with the carbonyl groups of the lipid. For the test compounds with longer alkyl chains, the hydrophobic interaction overpowers the polar groups’ interaction forcing the azido N_3_ group of the test compound deeper into the carbonyl region of the lipids.

**Figure 4:**
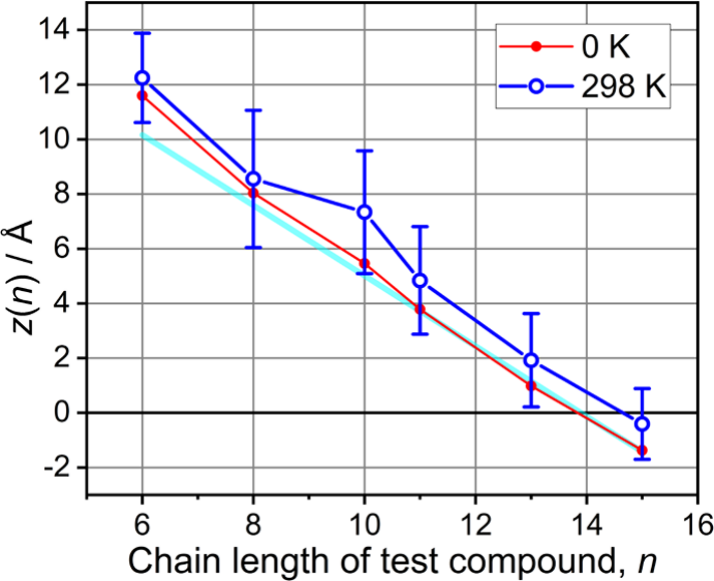
Depth of the azido moiety of the test compounds in the lipid bilayer MD vs the chain length, computed at 0 and 298 K. *z*(*n*) is measured as the distance between the central nitrogen atom of the N_3_ moiety and the plane separating the two leaflets (see the SI for details). The cyan line shows the slope of 1.286 Å per methylene unit of the chain.

To better understand the location (depth) of the azido group of the test compounds in the bilayer, we performed MD simulations. A fully hydrated lipid bilayer of 128 DPPC molecules, containing 1920 H_2_O molecules, was prepared using CHARMM-GUI.^76^ The simulations were performed using the NAMD software with the CHARMM36 force field.^77,78^ After 100 ps energy minimization and 1 ns thermalization, Langevin dynamics was run for 3 ns using a time step of 2 fs with constant temperature and pressure control. The points for the trajectories were recorded every 2 ps (see the SI for details). The *z*(*n*) dependence obtained at a minimization temperature of 0 K is shown with red circles in Figure 4. A cyan line shows the expected N_3_ depth assuming that the carboxylic acid moieties are aligned at the same depth with respect to the carbonyl groups of the lipids and taking an all-anti-conformation of the test compound chains. We see that at longer chains, *n* ≥ 11, the 0 K data have the same slope as the cyan line, indicating that their COOH groups are located at the same depth. The test compounds with shorter chains, especially those with *n* = 6, move up in the bilayer, as expected by reduced stabilization energy of the hydrophobic interaction for shorter chains. The MD data obtained at 298 K temperature, blue circles in Figure 4, show trends similar to those of 0 K data. Overall, the MD simulations support the location of the N_3_ moieties of the test compounds, as expected, if their COOH groups are located at approximately a constant depth in the bilayer.

### 3.3 2DIR Spectral Diffusion Measurements at Room Temperature

**Figure 5:**
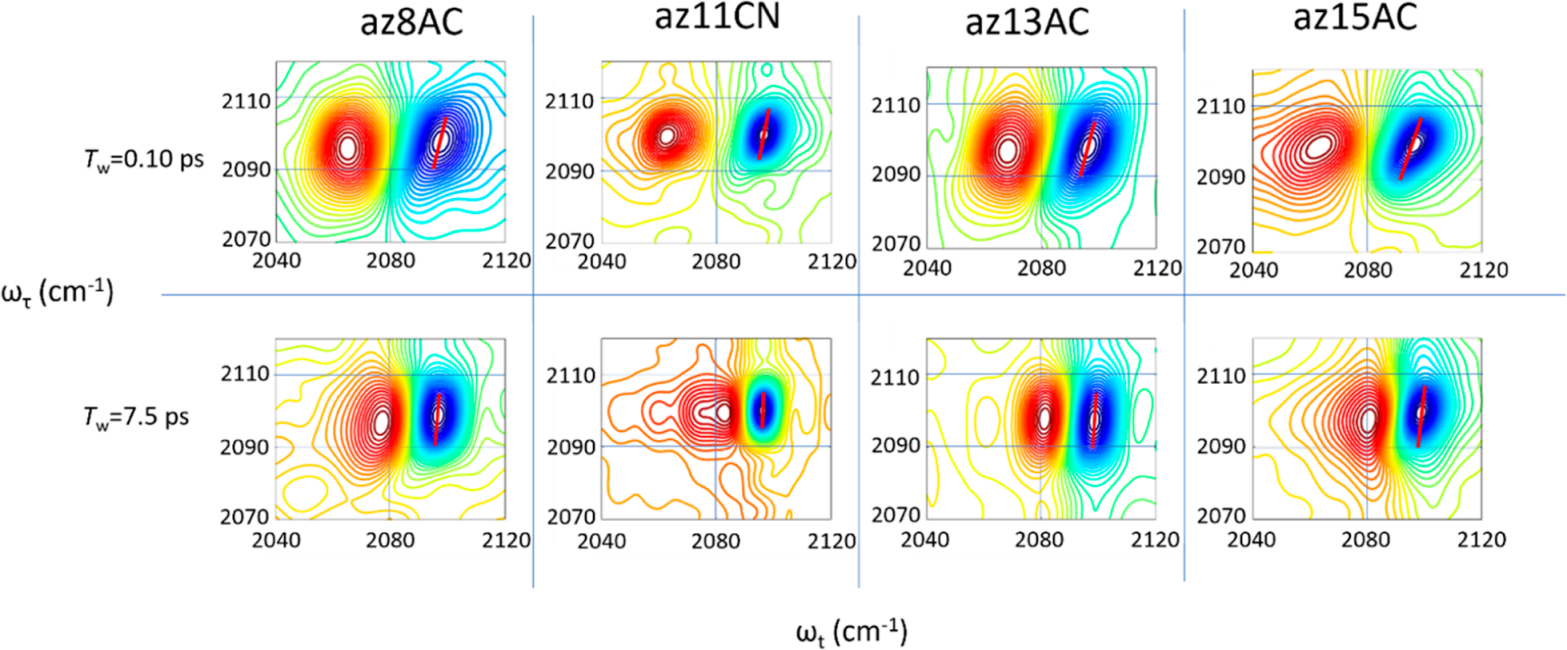
Absorptive 2DIR spectra of different test compounds at waiting times of 0.1 ps (top row) and 7.5 ps (bottom row) at 25 °C.

**Figure 6:**
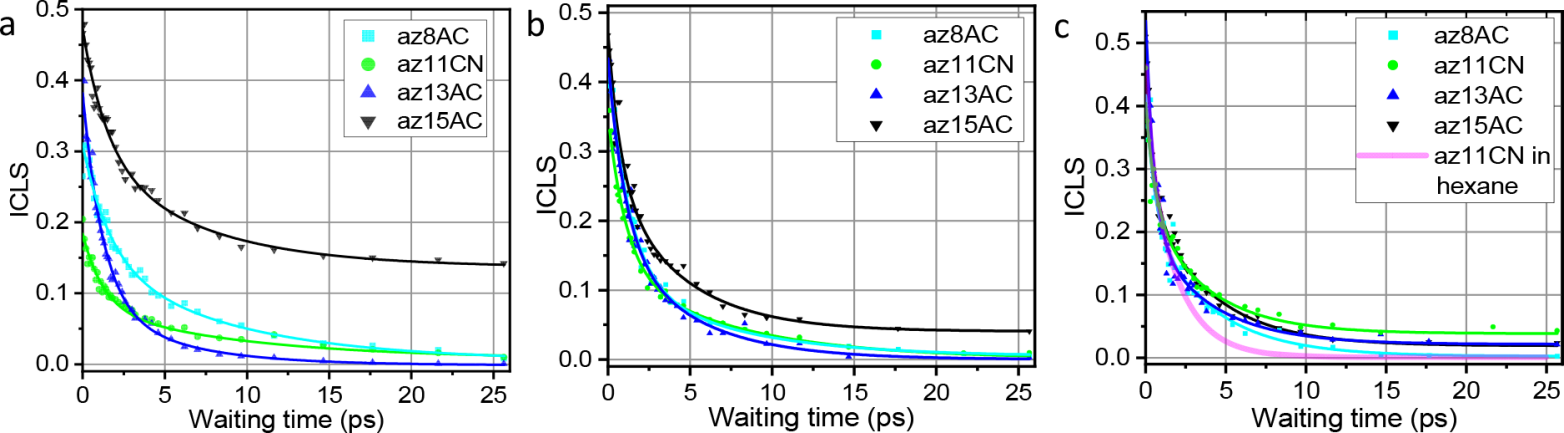
Waiting time dependences of the inverse center line slope of ν_N3_ for the indicated compounds in DPPC bilayers at (a) 25 °C, (b) 35 °C, and (c) 45 °C. Solid lines show fits with a double-exponential function (Tables S1–S3). The magenta line in panel c shows the fit of the data for az11CN in hexane measured at 25 °C, which was reprinted from ref (58) with permission from Elsevier.

We used a 2DIR spectral diffusion method to characterize the mobility of the bilayer environments of the N_3_ label in each test compound. Figure 5 shows the absorptive 2DIR spectra for ν_N3_ diagonal peaks of four test compounds in the bilayer. The top row corresponds to the spectra at *T*_w_ = 0.1 ps, and the bottom row is for *T*_w_ = 7.5 ps. The blue and red peaks correspond to 0 → 1 and 1 → 2 vibrational transitions of ν_N3_, respectively. The red line indicates the center line for the 0 → 1 peak, which is obtained by connecting the minima of the slices parallel to the *ω*_*t*_ axis. The inverse slope of this line, the inverse center line slope (ICLS), is plotted as a function of waiting time (Figure 6), which reports directly on the structural dynamics of its local environment.^79,80^

The 2DIR spectral diffusion approach relies on the presence of inhomogeneity for a vibrational mode. Due to inhomogeneity, different subsets of conformations are excited at their respective frequencies at the time of excitation, all within the peak line width.^81^ At zero waiting time, these excited subsets are observed at the same frequencies they possessed when excited, leading to diagonal elongation of the 2DIR peak of the mode. However, at later waiting times, the N_3_ group environment changes, causing the mode frequency to change from its original value at the time of excitation. Therefore, at larger waiting times the diagonal peak becomes less diagonally elongated and, eventually, when the memory of the conformations at the moment of excitation is lost, fully circular. ICLS reports on the frequency–frequency correlation function (FFCF) associated with the dynamics of the environment.^79,80^ Note that the FFCF represents the average probability of each frequency component of an inhomogeneous distribution to maintain the same frequency as a function of time.

**Figure 7:**
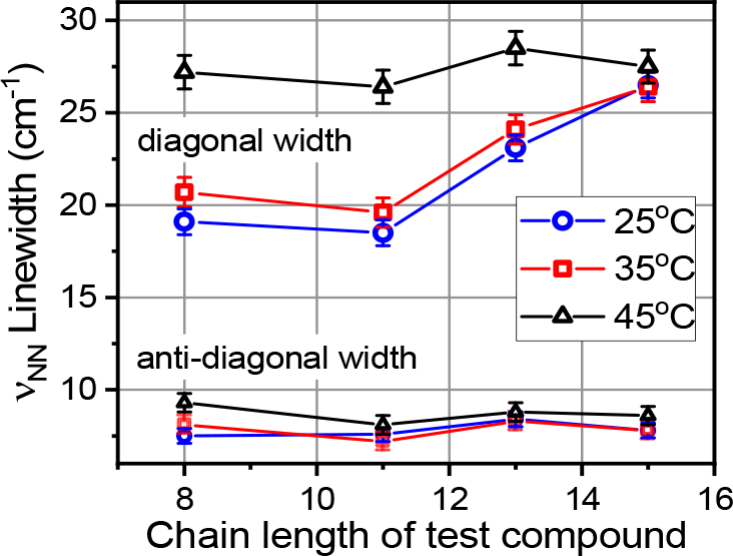
Homogeneous (antidiagonal) and inhomogeneous (diagonal) line widths (fwhm) of ν_N3_ in different test compounds as a function of the chain length at different temperatures.

Figure 6a shows the waiting-time changes in ICLS for four compounds in DPPC at 25 °C. The experimental data (points) were fitted with a double-exponential decay function (lines). The traces differ significantly for different compounds featuring different depths of the N_3_ label. First, notice that the initial ICLS values at *T*_w_ = 0 monotonically follow the width of the transitions in the FTIR spectrum (Figure S3). The initial ICLS is the smallest for az11CN and the largest for az15AC, indicating that the difference in the N_3_ peak width is due to different inhomogeneous contributions at different depths in the bilayer. To confirm this statement, the ellipticity of the diagonal peaks at zero waiting times was analyzed. The inhomogeneous width was obtained from the elongation of the diagonal peak along the diagonal direction in the rephasing spectra, whereas the homogeneous width was determined from its width in the antidiagonal direction (Figure S4).^82−84^ The results show that the antidiagonal width is similar for all the compounds (Figure 7, Tables S1–S3), while the diagonal width varies greatly, thus confirming that the difference in the width, observed in the FTIR measurements (Figure 1), is almost entirely due to the changes in the inhomogeneity the label experiences at different depths in the bilayer.

The spectral diffusion kinetics, shown in Figure 6a, differ significantly, reporting on the mobilities of the hydrophobic environments at the respective depths (Table S1). Several drastic differences are discussed below. The spectral diffusion dynamics for the az15AC compound is largely incomplete at 25 ps, showing a plateau. We hypothesize that the N_3_ label of az15AC is intercalated into the opposing leaflet. Such intercalation may result in a broader range of θ angles, leading to a broader spectrum. It can also lead to slower structural randomization of intercalated N_3_ groups, as they may require a motion of lipid molecules in opposing leaflets, which is less local. Thus, the experimental data support the proposed location of the N_3_ label of az15AC at the interface between the leaflets with some intercalation into the opposing leaflet.

The spectral diffusion decay times for compounds az8AC, az11CN, and az15AC show similarities to the mean time of ca. 4.5 ps (Table S1). The spectral diffusion decay for az13AC is significantly faster than those for other test compounds with a mean time of ca. 2.2 ps (Table S1). It is likely reflecting the known high fluidity of the region between two leaflets and also suggesting that no intercalation of the N_3_ group into the opposite leaflet takes place for az13AC. Measurements for a denser grid of chain lengths of the test compounds may shed light on the reasons for the kinetics differences.

With an increase in the temperature, the dynamics for all of the test compounds become faster (Figure 6b,c). At temperatures higher than *T*_ph_, the ICLS(*T*_w_) traces for different test compounds became similar to each other (Figure 6c), indicating that the structural order of the lipid chains present in the gel phase is essentially eliminated at all depths in the bilayer. The fast component of the ICSL decays of 0.4–0.6 ps is close to the fast component of the ICLS decay in hexane solvent, measured for az11CN at room temperature (Figure 6c, magenta line). Nevertheless, the ICLS decays for all compounds show a slower long component, of ca. 3.8 ps, compared to 1.9 ps in hexane. The plateau levels in Figure 6c feature errors of ca. ±0.04, which make their presence uncertain within the error bars.

Interestingly, already at 35 °C, which is below the phase transition, the labels in all four compounds show a significant increase in the mobility of their environments (Figure 6b). The decrease of the mean decay time of ca. 1.4-fold is found for az11CN and az15AC. A large increase in the apparent mobility is observed in az8AC (ca. 1.8-fold), which is likely affected by the broader depth distribution of its label. A much smaller value of the plateau is observed for az15AC at 35 °C, compared to that at 25 °C. The observed changes of the ICLS dynamics at the temperatures below *T*_ph_ demonstrate the high sensitivity of the N_3_ label to the mobility of the environment. The available dynamic range of the measured ILCS dynamics depends on the signal-to-noise (SN) level of the data. While the current SN level can be significantly improved, the reported data already feature a dynamic range of ca. 10-fold. Figure 7 summarizes the inhomogeneous and homogeneous width findings for the N_3_ labels at different depths and different temperatures.

### The Origin of Inhomogeneity

**Figure 8:**
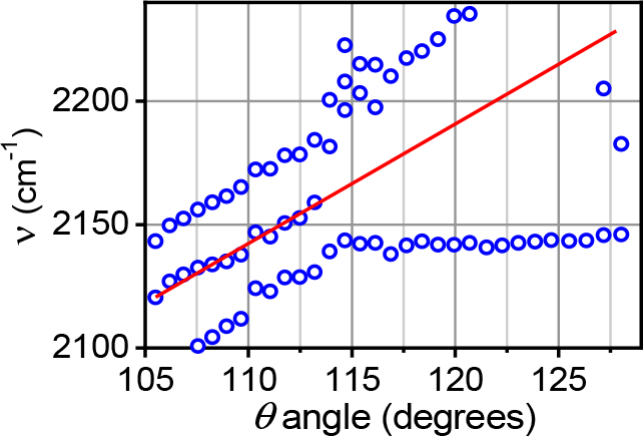
Frequencies of Fermi-coupled states, DFT-computed for az-(CH_2_)_5_-T for every θ angle. Here T stands for a heavy hydrogen atom assigned the atomic mass of 1000 u. The computational details are given in ref (58). The slope of the red line, given as a guide to the eye, is ca. 5 cm^–1^/deg, indicates a strong internal inhomogeneity associated with the θ angle. Reprinted from ref (58) with permission from Elsevier.

For a typical IR label, the inhomogeneous broadening increases with an increase of the polarity of the medium, and the alkyl-attached azido label shows such increase in polar solvents as well.^58^ However, what differentiates the N_3_ label from most other IR labels is a large inherent inhomogeneity dominated by internal degrees of freedom, mostly associated with the distribution of allowed θ angles. Figure 8 shows the frequencies of the computed Fermi-coupled states. These combination-band states borrow intensity from the ν_NN_ fundamental transition because of a large third-order coupling (Fermi resonance coupling) of ca. 30 cm^–1^.^72^ This intensity borrowing is the reason for the large width of the ν_N3_ peak width even in nonpolar solvents. As clear from Figure 8, the central frequency depends on θ in a monotonic fashion, increasing with θ at the rate of ca. 5 cm^–1^ per degree; see red line as a guide to the eye. As a result, the ν_N3_ peak shows inhomogeneous broadening that does not rely on the polarity of the environment, showing strong inhomogeneity even in nonpolar environments.

The presence of internal anharmonicity was observed in other compounds, such as metal carbonyls, experiencing soft potential for rotation around a single bond.^49,85^ However, in those examples, the induced inhomogeneity due to conformational distribution along the soft potential was rather small. The N_3_ group is unique, showing a very large internal inhomogeneity of ca. 30 cm^–1^, which leads to broad absorption peaks in nonpolar solvents (Figure S2). We determined that the θ angle is the main parameter causing the inhomogeneity, resulting in high sensitivity of the ν_N3_ peak width to restrictions in the θ angle distribution. When in a bilayer, such restrictions are caused by ordered microcrystalline fragments of the lipid chains. That is why the N_3_ peak width and the spectral diffusion dynamics are very sensitive to the temperature-induced phase transition in DPPC. Moreover, it responds well to the changes of temperature prior to undergoing the phase transition. Therefore, the ν_N3_ peak width reports on the width of the θ angle distribution, while the spectral diffusion dynamics reports on how quickly the environment is allowing the θ angle to change.

Note that the homogeneous width increases with temperature (Figure 7, antidiagonal width), although the increase is small. The homogeneous line width is expected to be dependent on temperature as *T*^1/2^, where *T* is the absolute temperature.^86,87^ However, within the narrow temperature region from 25 °C (298 K) to 45 °C (318 K), the dependence could appear as linear, as for az11CN in the hexadecane solvent. More detailed experiments are required to characterize different contributions to the line width increase in hexadecane.

### Order Parameter Associated with N_3_ Properties

The packing order inside lipid bilayers is customarily characterized by the order parameter, |*S*|, which reports on the deviation of the mean C–H bond direction from the normal to the bilayer and thus is the structural measure of the alignment of hydrocarbon chains in the membrane. Experiments and MD calculations show that the order parameter is the highest around the eighth carbon for the saturated lipid chains. The order decreases slightly toward the carbonyl groups and decreases sharply toward the middle of the bilayer.^17,29,30,88,89^ Note that the depth profiles of the order parameter, |*S*|, are similar at temperatures above and below *T*_ph_.

**Figure 9:**
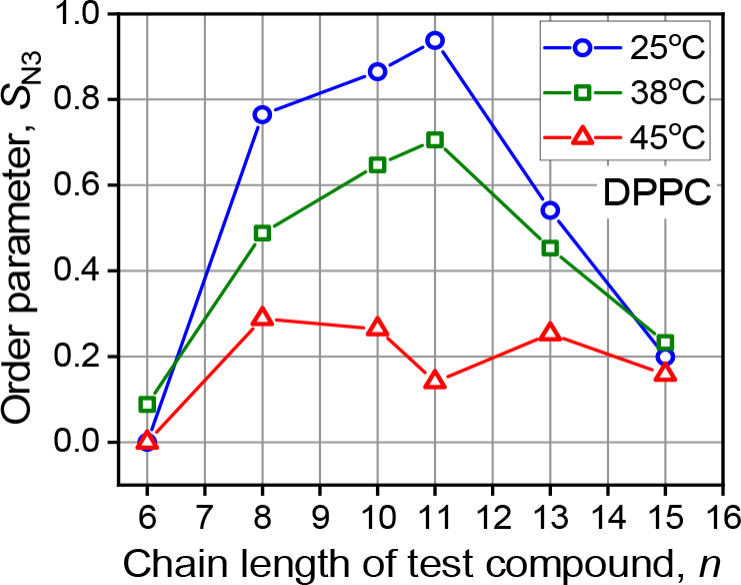
Dependence of the *S*_N3_ order parameter on *n* for the three temperatures.

Based on the width of ν_N3_ reporter in the bilayer, we introduced an order parameter as , where *δν̅*_max_ and *δν̅*_min_ are the largest and smallest N_3_ peak widths among all N_3_ locations in the bilayer interior and *δν̅*_*n*_ is the actual peak width of the test compound with the chain length *n*. The smallest peak width was observed for az11AC (Figure 3b). Importantly, the peak width is temperature dependent and was showing the width of ca. 14 cm^–1^ at 22 °C, which was taken as *δν̅*_min_. The largest width, when in the bilayer, *δν̅*_max_, was taken as 31 cm^–1^, matching the width for az6AC where the N_3_ reporter is located in the vicinity of carbonyl groups of the bilayer. Figure 9 shows the *n* dependence of the order parameter, *S*_N3_, at three temperatures (see also Figure S5). The curves depend strongly on temperature and, at temperatures below *T*_ph_, peak at *n* = 10–11. The peak of *S*_N3_ occurs at longer depths, compared to that of |S|, indicating that although the chain becomes more “tilted” at the depth of 10–11 (|*S*|), it nevertheless provides the most rigid environment for the motion.

Note that the structural order parameter, |*S*|, assessed from the C–H bond angle distribution, is different from the mobility parameter introduced here for the N_3_ label properties. The following example illustrates the difference. Suppose the lipid chains surrounding the N_3_ group have gauche kinks, which make the chains structurally disordered (the NMR order parameter, |*S*|, is low). However, if the neighboring lipid chains follow the same chain shape, the mobility of such a well-packed environment is expected to provide constraints for motion, which will be reported by the N_3_ label as a “constraint”, nonmobile environment. This example illustrates that the N_3_ group measures the structural restrictions imposed by the environment and not just the orientational disorder of the lipid chains. In fact, the N_3_ label is capable of measuring both the extent of structural disorder of the environment via the width of its θ-angle distribution as well as the time-resolved dynamics of the environment using spectral diffusion.

## Conclusions

We designed and synthesized test compounds to study the lipid bilayer interior with high spatial and temporal resolution. The test compounds consist of an azido group tethered by an alkyl linker to a polar group, such as a carboxylic acid or cyano group. Using FTIR and 2DIR spectroscopies, we have demonstrated that when in the bilayer, the polar group of the compound is in the vicinity of the carbonyl groups of the lipids, while the azido-group reporter is located in the nonpolar section of the bilayer at the depth that is dependent on the length of the alkyl chain. We found that the properties of the IR reporter, the asymmetric stretching mode of N_3_, such as the absorption peak width, inhomogeneity, and spectral diffusion dynamics, change sensitively with the depth of the N_3_ group in the bilayer, reporting on how the bilayer properties change with the depth in the bilayer. The local environment mobilities at different depths in the bilayer were investigated as a function of temperature. When the N_3_ group is placed in the middle of a leaflet, alkyl group lengths of 8–13 carbons, the temperature-induced phase transition is well observed, showing the S-like temperature dependence. Based on the N_3_ group properties, we introduced an order parameter, *S*_N3_, which characterizes the restrictions to motion inside the bilayer. It shows strong sensitivity to the depth in the bilayer and to temperature. The order parameter is the highest at *n* = 10–11 and drops for smaller *n* (toward the head groups) and larger *n* toward the middle of the bilayer. It shows a strong sensitivity to the phase transition. Analysis of the diagonal 2DIR peaks of N_3_ showed different levels of inhomogeneity at different depths in the bilayer, with the smallest inhomogeneity in the middle of a leaflet. The spectral diffusion dynamics, determined from the center line slope analysis for the N_3_ peak, were also found to be dependent on the depth of the N_3_ group in the bilayer at room temperature. Moreover, at temperatures above the phase transition (*T* > 41 °C), the dynamics became faster and much less dependent on the detection depth, indicating higher fluidity of the bilayer. The study has proven the high sensitivity of the N_3_ label for studying bilayer mobility at different depths. It showed that the bilayer properties are significantly different in the middle of the bilayer and in the middle of leaflets, opening avenues for further studies of various hydrophobic interiors in bilayers and beyond.

It would be interesting to investigate the variations in crystallinity and dynamics of a bilayer due to other membrane components such as cholesterol, other lipid types, and proteins. Note that a large number of processes taking place in cell membranes are site and depth specific. The method developed in this study can be used to undertake a range of biochemistry questions, including membrane permeability and membrane reaction mechanisms. When positioned at a specific depth, the azido group can be used to track other components of the bilayer via 2DIR cross-peak measurements. Note that N_3_ can also form hydrogen bonds with other components of a bilayer, in which case the knowledge of its depth collation could be useful. Finally, the described measurements can serve to test the accuracy of molecular dynamics simulations, which will increase our understanding of cell membrane dynamics. The developed assay is rather simple and readily applicable to the study of various bilayers and cell membranes.

## Supporting Information Available

Additional figures and details of 2DIR measurements, synthesis, sample preparation, and MD simulations (PDF)
