# Supplementary material for: Probing the Hydrophobic Region of a Lipid Bilayer at Specific Depths Using Vibrational Spectroscopy
Source: J Am Chem Soc. 2023 Nov 20;145(48):26363–73. doi: 10.1021/jacs.3c10178 (PMC10704553; doi:10.1021/jacs.3c10178)
Supplement: Supplementary file 1 — ja3c10178_si_001.pdf [file ja3c10178_si_001.pdf]

Supplementary Materials

Probing Hydrophobic Region of a Lipid Bilayer at Specific Depths Using Vibrational Spectroscopy

Md Muhaiminul Islam, Sithara U. Nawagamuwage, Igor V. Parshin, Margaret C. Richard, Alexander L. Burin, and Igor V. Rubtsov*

Department of Chemistry, Tulane University, New Orleans, Louisiana 70118, United States

Table of contents

1. Additional figures and data.
2. Details of 2DIR spectroscopy measurements.
3. Preparation of planar multilamellar bilayer samples
4. Molecular dynamics simulations.
5. Synthesis of az*n*AC compounds.

1. Additional figures

Phase transition in DPPC bilayer measured using a CH_2 stretching mode at ca. 2850 cm^{-1} (Fig. S1).

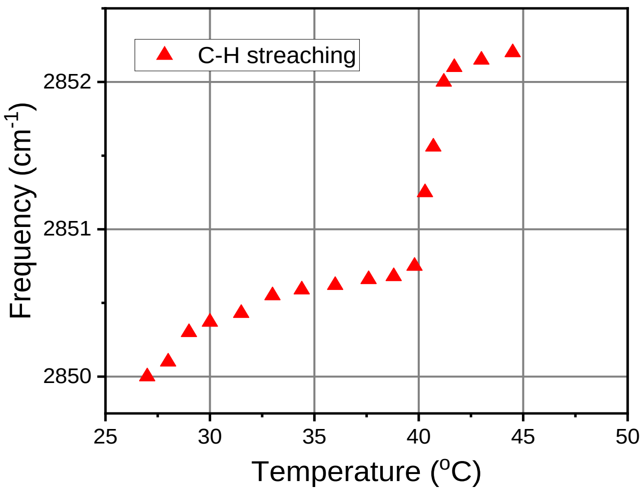

Fig. S1. DPPC Lipid phase transition measured using CH_2 stretch peak at ca. 2850 cm^{-1}. Note that the CH_2 stretching modes of the lipids report on the depth-averaged ordering of the lipid bilayer.

The ν_{N3} width for az11CN in two solvents shows a slight increase with temperature (Fig. S2).

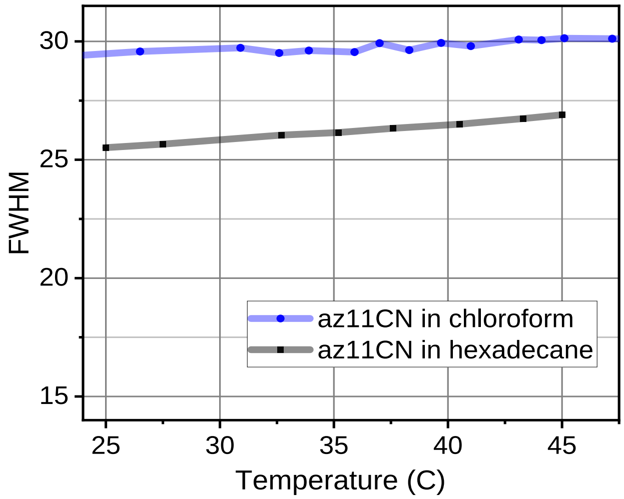

Fig. S2. ν_{N3} peak width for az11CN in chloroform and hexadecane as a function of temperature.

The initial ICLS obtained at $T_w = 0$ (Fig. S3, blue circles) follows monotonically the width of the transition in the FTIR spectrum (peak width), indicating that the difference in N_3 peak width is caused by different inhomogeneous contributions the N_3 label experienced at different depths in the bilayer.

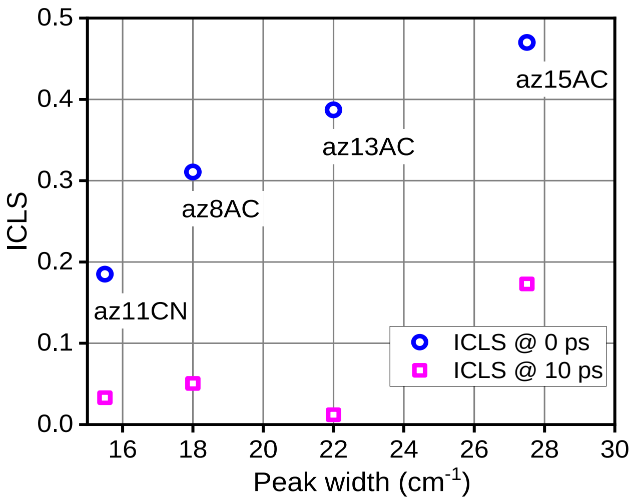

Fig. S3. ICLS obtained at T_w = 0 (blue circles) and T_w = 10 ps (magenta squares) for indicated compounds.

The inhomogeneous and homogeneous widths of the N_3 transition were evaluated from diagonal and antidiagonal width of the rephasing 2DIR spectrum (Fig. S4).

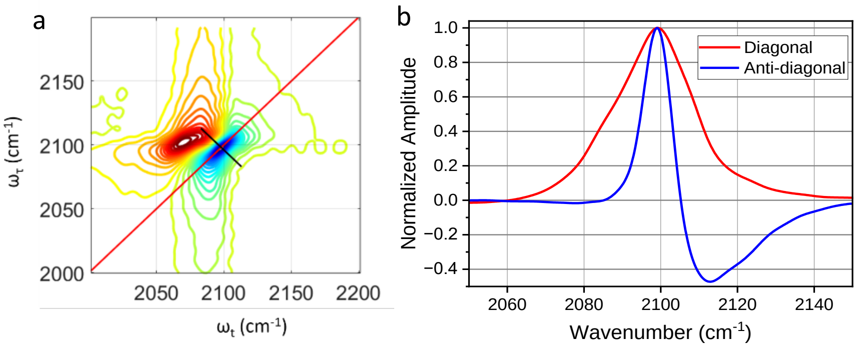

Fig. S4. a. Rephasing 2DIR spectrum of az11AC in DPPC bilayer, measured at T_w =0.2 ps and 25 ºC. b. The diagonal and antidiagonal cross sections of the 2DIR spectrum in panel a.

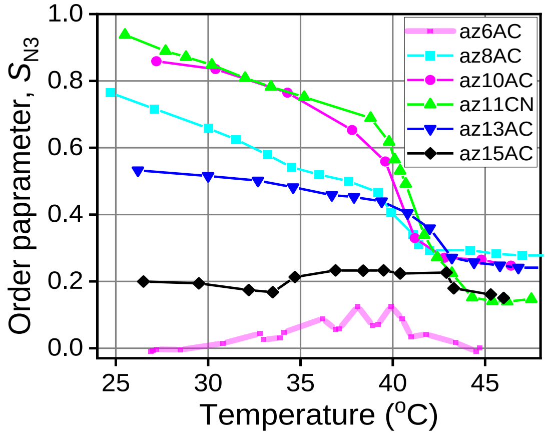

Fig. S5. Dependence of S_{N3} on temperature for different test compounds.

Fitting of spectral diffusion data with a double-exponential function.

Table S1. Results of fitting of waiting time dependence of ICLS measured at 25ºC with a double-exponential function. Diagonal and antidiagonal widths are also shown.

25º C	y_0	τ_1, ps (A_1, %)	τ_2, ps (A_2, %)	τ_{mean} (ps)	Antidiagonal linewidth, cm^{-1}	Diagonal linewidth, cm^{-1}
az8AC	0.0006 ± 0.005	1.7 ± 0.2 (52%)	8.5 ± 1.5 (48%)	5.0	7.5 ± 0.4	19.1 ± 0.7
az11CN	0.009 ± 0.006	1.4 ± 0.1 (54%)	9.1 ± 0.9 (41%)	4.7	7.6 ± 0.4	18.5 ± 0.7
az13AC	0.002 ± 0.004	1.3 ± 0.1 (78%)	5.5 ± 0.8 (22%)	2.2	8.4 ± 0.4	23.1 ± 0.7
az15AC	0.13 ± 0.01	1.3 ± 0.1 (34%)	6.9 ± 0.4 (38%)	4.3	7.8 ± 0.4	26.5 ± 0.7

Table S2. Results of fitting of waiting time dependence of ICLS measured at 35ºC with a double-exponential function. Diagonal and antidiagonal widths are shown.

35ºC	y_0	τ_1, ps (A_1, %)	τ_2, ps (A_2, %)	τ_{mean} (ps)	Antidiagonal linewidth, cm^{-1}	Diagonal linewidth, cm^{-1}
az8AC	0.004 ± 0.002	1.3 ± 0.1 (71%)	6.7 ± 1.2 (28%)	2.8	8.1 ± 0.5	20.5 ± 0.8
az11CN	0.001 ± 0.001	1.1 ± 0.1 (62%)	7.4 ± 0.6 (37%)	3.4	7.2 ± 0.5	19.6 ± 0.8
az13AC	4E-4 ± 0.001	1.1 ± 0.1 (58%)	4.6 ± 0.5 (42%)	2.5	8.3 ± 0.5	24.1 ± 0.8
az15AC	0.040 ± 0.02	0.79 ± 0.15 (44%)	4.3 ± 0.6 (49%)	2.6	7.8 ± 0.5	26.4 ± 0.8

Table S3. Results of fitting of waiting time dependence of ICLS measured at 45ºC with a double-exponential function. Diagonal and antidiagonal widths are shown.

45ºC	y_0	τ_1, ps (A_1, %)	τ_2, ps (A_2, %)	τ_{mean} (ps)	Antidiagonal linewidth, cm^{-1}	Diagonal linewidth, cm^{-1}
az8AC	0.0006 ± 0.003	0.56 ± 0.1 (44)	3.9 ± 0.7 (56)	2.4	9.3 ± 0.5	27.2 ± 0.9
az11CN	0.009 ± 0.002	0.60 ± 0.1 (17%)	3.5 ± 0.2 (73%)	2.9	8.1 ± 0.5	26.4 ± 0.9
az13AC	0.002 ± 0.002	0.51 ± 0.05 (65%)	3.8 ± 0.4 (32%)	1.6	8.8 ± 0.5	28.5 ± 0.9
az15AC	0.024 ± 0.003	0.41 ± 0.05 (53%)	3.9 ± 0.4 (43%)	2.0	8.6 ± 0.5	27.5 ± 0.9

2. Details of 2DIR spectroscopy measurements.

The instrument and method for a fully automated dual-frequency three pulse photon echo 2DIR spectrometer with heterodyned detection has been described elsewhere.(1, 2) To summarize, the laser pulses at 800 nm and 1 kHz (Libra, Coherent) repetition rate were used to pump two optical parametric amplifiers (OPA) (Palitra, Quantronix), followed by two difference-frequency generation (DFG) units. The instrument features built-in beam direction stabilization accurate to 50 μrad, closed-loop phase stabilization accurate to 70 as, phase cycling, and automatic tuning of the phase-matching beam geometry. The three mid-IR pulses had pulse energies of ca. 1 μJ and their polarizations were parallel. The beam diameter in the sample cell was approximately 100 μm. The absorptive 2DIR spectra were obtained as a sum of the real part rephasing and non-rephasing 2DIR spectra, phased individually based on the pump-probe spectra of the respective transition. The center line slopes (CLS) in the absorptive 2DIR spectra were determined from the line connecting peak position of the one-dimensional cuts parallel to the pump axis at the ground-state bleach/stimulated emission (GSB/SE) peaks. Note that the bending-libration combination band of water, observed at approximately 2150 cm^{-1} and located close to the studied azido-group peak, did not appear in the 2DIR spectra in MLBL due to its small extinction coefficient and very large width.

3. Preparation of planar multilamellar bilayer samples

Planar multilamellar bilayer samples used for the measurements were prepared by Isopotential spin-dry ultracentrifugation (ISDU) method adopted from the work of Freed et al. (3, 4) A test compound was mixed with di-palmitoyl phosphatidylcholine (DPPC, Avanti Polar Lipids) at ca. 1:10 molar ratio in chloroform. The resulting mixture was kept in vacuum overnight to remove the solvent. Then the dry lipid film was dissolved in water, followed by sonication at temperature above the phase transition temperature.

The sample, containing unilamellar vesicles was placed into an in-house designed cell over a CaF_2 window (12 mm in diameter and 1 mm thick) for ultracentrifugation. The ultracentrifugation was performed at ca. 40,000 g at 20°C for 18 hours to slowly evaporate the water. The procedure produced an optically clear and highly aligned multilamellar planar bilayer (MLBL) on the CaF_2 wafer, which resulted in approximately 2 water molecules per lipid. The bilayer sample was hydrated by adding ca. 1 µL of H_2O and sandwiched between two CaF_2 windows and sealed in an in-house made cell holder. The water content in the bilayer was monitored by observing water peaks in the FTIR spectrum. The water content was maintained at a constant level of 13-15 water molecules per lipid; the same results were found with the water content exceeding 10 water molecules per lipid. To ensure precise temperature control during the FTIR and 2DIR measurements, an insulating jacket was used to enclose the sample cell, which was equipped with a homemade temperature controller. The temperature was kept constant (± 0.2 °C) throughout the measurements and monitored using a thermocouple that was attached to the CaF_2 substrate of the sample cell.

The alignment of the planar bilayer sample was confirmed using cross polarizers. The unaligned bilayer is known to rotate the polarization of the incident beam.(5, 6) Two polarizers with polarization perpendicular to each other was placed before and after the sample and was placed in the FTIR. The sample did not rotate the polarization of incident IR beam and appeared blank. Furthermore, FTIR spectra of different regions of the sample was measured and were found to be the same. The molecular volume of the test compounds is substantially smaller than that of DPPC molecules as each DPPC molecule has 2 tails and a large head group.

4. Molecular dynamics simulations.

Molecular dynamics simulations were performed for a fully hydrated lipid bilayer of 128 dipalmitoyl phosphatidylcholine (DPPC) molecules generated with CHARMM-GUI Software.(7) The guest molecule (az*n*AC) was added using Avogadro Software (version 1.2.0),(8) resulting in a molecular system with 128 lipid molecules, 1920 water molecules, and one guest molecule (Figure S6). All simulations were run with NAMD software using CHARMM36 force field for lipids (9, 10) and the CHARMM TIP3P water model.(11, 12) Hydrogen atoms were resolved using psfgen plugin of NAMD.(13) We used recently reported CHARMM36 FF parameters for the azido group, calculated by Smith and co-workers.(14)

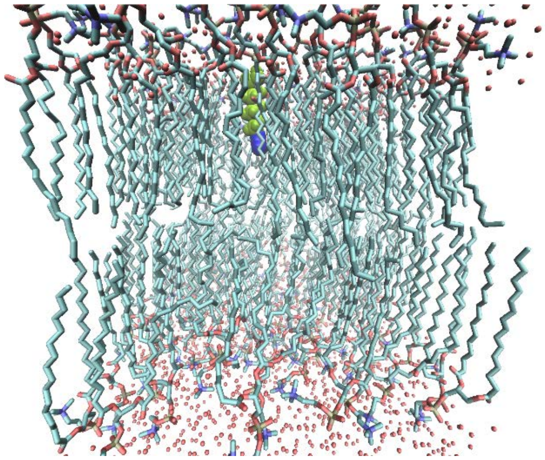

Figure S6. Molecular system (lipid bilayer, water and az13AC guest molecule) used for the MD simulations.

System energy minimization was run for 100 ps to attain the energy minimum, then the system was heated up to the room temperature (298 K) and molecular dynamics was run for 3 ns with a timestep of 2 fs in NPT ensemble after 1 ns thermalization. Langevin damping factor of 1ps^{-1} was introduced for constant temperature control. Langevin piston (target pressure of 0.01 bar, piston period of 200 fs and a piston decay

of 100 fs) was used for reinforcing tighter packing of the bilayer. Trajectories were collected every 2 ps. The depth of the N_3 moiety in the bilayer was characterized as an average distance between the middle nitrogen atom of N_3 and the middle plane separating the two leaflets of the bilayer.

5. Synthesis of az*n*AC compounds.

In short, sodium azide was added to $BrC_nH_{2n}COOH$ or $BrC_nH_{2n}CN$ at 1.5:1 mole ratio in anhydrous DMF. The mixture was stirred for 12 hours at 60°C. Then the resultant mixture was diluted with hexane and washed with brine. The organic layer was separated and dried over Na_2SO_4. Finally, solvent was removed with a rotavap, yielding the product. In all cases >95% yield was found. The product was verified by FTIR and NMR. The following ^{1}H NMR (400 MHz, $CDCl_3$) data were obtained: 7-azidoheptanoic acid: δ 3.28 (t, j=6.9 Hz 2H), 2.38 (t, j=7.4 Hz, 2 H), 1.76-1.50 Hz (m, 4 H), 1.50-1.26 Hz (m, 4 H); 7-azidoheptanenitrile: δ 3.28 (t, j=6.9 Hz 2H), 2.38 (t, j=7.6 Hz, 2 H), 1.78-1.52 Hz (m, 4 H), 1.50-1.28 Hz (m, 4 H); 9-azidononanoic acid: δ 3.27 (t, j=6.8 Hz 2H), 2.37 (t, j=7.4 Hz, 2 H), 1.71-1.49 Hz (m, 4 H), 1.44-1.21 Hz (m, 8 H); 11-azidoundecanoic acid: δ 3.27 (t, j=6.9 Hz 2H), 2.36 (t, j=7.5 Hz, 2 H), 1.71-1.53 Hz (m, 4 H), 1.50-1.40 Hz (m, 4 H), 1.44-1.23 Hz (m, 12 H); 12-azidododecanenitrile: δ 3.27 (t, j=6.9 Hz 2H), 2.35 (t, j=7.2 Hz, 2 H), 1.75-1.53 Hz (m, 4 H), 1.50-1.40 Hz (m, 4 H), 1.40-1.22 Hz (m, 10 H); 14-azidotetradecanoic acid: δ 3.28 (t, j=7.0 Hz 2 H), 2.37 (t, j=7.5 2 H), 1.70-1.57 Hz (m, 4 H), 1.42-1.22 Hz (m, 18 H); 16-azidohexadecanoic acid: δ 3.28 (t, j=6.9 Hz 2 H), 2.37 (t, j=7.6 2 H), 1.72-1.55 Hz (m, 4 H), 1.45-1.23 Hz (m, 22 H).

References

(1) Leger, J.; Nyby, C.; Varner, C.; Tang, J.; Rubtsova, N. I.; Yue, Y.; Kireev, V.; Burtsev, V.; Qasim, L.; Rubtsov, G. I.; Rubtsov, I. V. Fully automated dual-frequency three-pulse-echo 2DIR spectrometer accessing spectral range from 800 to 4000 wavenumbers. *Rev. Sci. Instr.* **2014**, *85*, 083109. DOI: 10.1063/1.4892480.

(2) Nyby, C. M.; Leger, J. D.; Tang, J.; Varner, C.; Kireev, V. V.; Rubtsov, I. V. Mid-IR beam direction stabilization scheme for vibrational spectroscopy, including dual-frequency 2DIR. *Opt. Express* **2014**, *22* (6), 6801-6809. DOI: 10.1364/OE.22.006801.

(3) Dzikovski, B.; Tipikin, D.; Freed, J. Conformational Distributions and Hydrogen Bonding in Gel and Frozen Lipid Bilayers: A High Frequency Spin-Label ESR Study. *The Journal of Physical Chemistry B* **2012**, *116* (23), 6694-6706. DOI: 10.1021/jp211879s.

(4) Ge, M.; Budil, D. E.; Freed, J. H. ESR studies of spin-labeled membranes aligned by isopotential spin-dry ultracentrifugation: lipid-protein interactions. *Biophysical Journal* **1994**, *67* (6), 2326-2344. DOI: 10.1016/S0006-3495(94)80719-2.

(5) Kel, O.; Tamimi, A.; Thielges, M. C.; Fayer, M. D. Ultrafast Structural Dynamics Inside Planar Phospholipid Multibilayer Model Cell Membranes Measured with 2D IR Spectroscopy. *Journal of the American Chemical Society* **2013**, *135* (30), 11063-11074. DOI: 10.1021/ja403675x.

(6) Stevenson, P.; Tokmakoff, A. Ultrafast Fluctuations of High Amplitude Electric Fields in Lipid Membranes. *Journal of the American Chemical Society* **2017**, *139* (13), 4743-4752. DOI: 10.1021/jacs.6b12412.

(7) Patra, M.; Karttunen, M.; Hyvönen, M. T.; Falck, E.; Lindqvist, P.; Vattulainen, I. Molecular Dynamics Simulations of Lipid Bilayers: Major Artifacts Due to Truncating Electrostatic Interactions. *Biophysical Journal* **2003**, *84* (6), 3636-3645. DOI: 10.1016/S0006-3495(03)75094-2.

(8) Hanwell, M. D.; Curtis, D. E.; Lonie, D. C.; Vandermeersch, T.; Zurek, E.; Hutchison, G. R. Avogadro: an advanced semantic chemical editor, visualization, and analysis platform. *Journal of Cheminformatics* **2012**, *4* (1), 17. DOI: 10.1186/1758-2946-4-17.
(9) Klauda, J. B.; Venable, R. M.; Freites, J. A.; O'Connor, J. W.; Tobias, D. J.; Mondragon-Ramirez, C.; Vorobyov, I.; MacKerell, A. D., Jr.; Pastor, R. W. Update of the CHARMM All-Atom Additive Force Field for Lipids: Validation on Six Lipid Types. *The Journal of Physical Chemistry B* **2010**, *114* (23), 7830-7843. DOI: 10.1021/jp101759q.
(10) Venable, Richard M.; Sodt, Alexander J.; Rogaski, B.; Rui, H.; Hatcher, E.; MacKerell, Alexander D.; Pastor, Richard W.; Klauda, Jeffery B. CHARMM All-Atom Additive Force Field for Sphingomyelin: Elucidation of Hydrogen Bonding and of Positive Curvature. *Biophysical Journal* **2014**, *107* (1), 134-145. DOI: 10.1016/j.bpj.2014.05.034.
(11) Jorgensen, W. L.; Chandrasekhar, J.; Madura, J. D.; Impey, R. W.; Klein, M. L. Comparison of simple potential functions for simulating liquid water. *The Journal of Chemical Physics* **1983**, *79* (2), 926-935. DOI: 10.1063/1.445869.
(12) Durell, S. R.; Brooks, B. R.; Ben-Naim, A. Solvent-induced forces between two hydrophilic groups. *The Journal of Physical Chemistry* **1994**, *98* (8), 2198-2202. DOI: 10.1021/j100059a038.
(13) Phillips, J. C.; Hardy, D. J.; Maia, J. D. C.; Stone, J. E.; Ribeiro, J. V.; Bernardi, R. C.; Buch, R.; Fiorin, G.; Hénin, J.; Jiang, W.; et al. Scalable molecular dynamics on CPU and GPU architectures with NAMD. *The Journal of Chemical Physics* **2020**, *153* (4). DOI: 10.1063/5.0014475.
(14) Smith, A. K.; Wilkerson, J. W.; Knotts, T. A. I. V. Parameterization of Unnatural Amino Acids with Azido and Alkynyl R-Groups for Use in Molecular Simulations. *The Journal of Physical Chemistry A* **2020**, *124* (30), 6246-6253. DOI: 10.1021/acs.jpca.0c04605.
